# miR-149 Promotes Apoptosis of Ovarian Granulosa Cells Through Inducing Caspase 8 Expression

**DOI:** 10.3390/ani16172663

**Published:** 2026-08-25

**Authors:** Miaomiao Wang, Chenxu Wang, Min Lu, Xiaoyu Zhang, Yajie Gao, Tengteng Xu, Yong Liu

**Affiliations:** Anhui Province Key Laboratory of Embryo Development and Reproductive Regulation, Fuyang Normal University, Fuyang 236037, China

**Keywords:** miR-149, saRNA, Caspase 8, apoptosis, sow fertility

## Abstract

Follicular atresia constitutes a major constraint on sow reproductive performance. Aberrant miRNA expression is well documented to promote follicular atresia. However, precise identification of the functional miRNAs responsible for this process remains a formidable challenge. In this study, we integrated multiple transcriptome datasets and identified miR-149, a miRNA located within the quantitative trait loci (QTL) associated with litter size in sow. Notably, miR-149 was highly expressed in atretic follicles and ovaries of sow with low litter size. Subsequent in vitro culture of ovarian granulosa cells (GCs) combined with miR-149 overexpression assays revealed that miR-149 significantly promotes the expression of cleaved caspase 3 (c-caspase 3), a marker of apoptosis in ovarian GCs. Mechanistic studies revealed that miR-149 functions as small activating RNA (saRNA) to activate the transcription of Caspase 8. This triggers the death receptor-mediated apoptotic, upregulates the expression of Caspase 3, and ultimately accelerates apoptosis in ovarian GCs. Collectively, these results indicate that miR-149 acts as negative regulator of sow reproduction. This study elucidates the miR-149/Caspase 8/Caspase 3 signaling axis as a novel pathway governing sow fertility, which provides molecular targets for improving reproductive performance in sow.

## Simple Summary

In this study, we identified and characterized a sow reproduction-related miRNA, miR-149, which was significantly upregulated in atretic follicles and ovarian tissues of sow with low litter size. We found that miR-149 activates the transcription of Caspase 8, thereby triggering the death receptor-mediated apoptotic, upregulates the expression of Caspase 3, and ultimately accelerates apoptosis in sow ovarian granulosa cells. These findings indicate that miR-149 as a detrimental regulator that impairs sow reproductive performance.

## 1. Introduction

In large-scale pig production, sow reproductive performance serves as a core indicator of production efficiency and economic benefits, which directly determines the overall production capacity of the pig industry. The normal growth, development, maturation, and ovulation of ovarian follicles, as well as a well-regulated follicular developmental cycle, constitute the physiological basis for high reproductive performance in sow. Follicular atresia, a prevalent physiological process during ovarian development, markedly reduces the number of viable ovulations and has become a major limiting factor that restricts the full expression of sow reproductive potential and further hinders the improvement of pig production capacity [1,2]. Currently, the pig industry is commonly plagued by inconsistent reproductive efficiency and unstable high-yield traits in sow, and aberrant regulation of follicular atresia is a critical internal cause underlying poor reproductive performance. Therefore, systematic elucidation of the molecular mechanisms governing follicular atresia and identification of key functional genes involved in follicular development and atresia are of great significance to the pig industry.

microRNAs (miRNAs) represent a group of non-coding RNAs around 22 nt in length. In their canonical regulatory mode, miRNAs bind to the 3′ untranslated region (UTR) of gene, thereby promoting the degradation of the target mRNA or inhibiting its translation [3,4,5]. Studies have demonstrated that miRNAs are extensively involved in multiple critical physiological processes, including follicular development, steroid hormone synthesis, as well as apoptosis of GCs [6,7,8,9]. Aberrantly expressed miRNAs in atretic follicles can directly drive follicular atresia by regulating expression of target genes. For instance, miR-361-5p directly targets the 3′UTR of VEGFA to promote GCs apoptosis and thereby accelerate follicular atresia [10]. miR-143 suppresses steroid hormone synthesis and induces apoptosis in GCs, consequently mediating follicular atresia [11]. In addition, miR-1275 blocks the LRH-1/CYP19A1 signaling pathway to modulate granulosa cell apoptosis and estradiol secretion, ultimately facilitating follicular atresia [12]. Nevertheless, most current studies screen candidate miRNAs solely based on single transcriptomic datasets and mainly focus on their canonical negative regulatory functions, while neglecting their non-canonical activating roles exemplified as small activating RNA (saRNA). This limitation compromises the accuracy of screening key miRNAs associated with sow fertility. Accordingly, it is necessary to integrate multi-source transcriptomic data combined with functional assays to systematically identify core miRNAs regulating sow fertility, particularly those exerting regulatory effects via non-canonical mechanisms.

In this study, transcriptomic datasets derived from ovarian tissues of sow with high and low litter size, as well as healthy and atretic follicles, were integrated together with sow reproductive trait QTL data. Finally, miR-149 was identified as one core candidate miRNA. This miRNA was highly expressed in both atretic follicles and ovarian of low-fertility sow. Nevertheless, its biological functions and underlying regulatory mechanisms involved in sow reproduction remain unclarified to date. Therefore, the present study aimed to identify the target genes of miR-149 and further elucidate its molecular mechanism underlying the modulation of sow fertility.

## 2. Materials and Methods

### 2.1. Bioinformatic Analysis

TargetScan (https://www.targetscan.org/vert_80/ (accessed on 11 June 2026)) and miRDB (https://mirdb.org/ (accessed on 11 June 2026)) were used to predict miR-149 target genes. The RNAhybrid was used to evaluate the binding of miR-149 to the Caspase 8 promoter and 5′ UTR regions. The DAVID (https://davidbioinformatics.nih.gov/ (accessed on 13 June 2026)) was used to perform enrichment analyses of the functions of potential miR-149 target genes and their associated signaling pathways.

### 2.2. Animals and Ethics

The ovaries from healthy, sexually mature sow were obtained from a local slaughterhouse for follicular isolation and ovarian GCs culture. Since the exact genetic relatedness among sows cannot be definitively determined, GCs collected from each batch of 100 sows’ ovaries (used across multiple experimental projects) were thoroughly mixed prior to inoculation. All animal procedures received approval from the Animal Ethics Committee of Fuyang Normal University (Fuyang, China).

### 2.3. Follicle Isolation and Classification

The fresh ovaries were washed several times with 37 °C saline solution and then placed in petri dish. The follicles were carefully isolated using a scalpel and forceps. The follicular membrane was gently ruptured in a separation column, and the follicular fluid was collected after centrifugation at 1000 rpm for 30 s. The levels of progesterone (P4) and 17β-estradiol (E2) in the follicular fluid were measured using an ELISA assay (North Biotech, Beijing, China). The health of the follicles is assessed based on the P4/E2 ratio: healthy follicles (HF): P4/E2 < 5; early atresia follicles (EAF): 5 ≤ P4/E2 ≤ 20 [13].

### 2.4. Cell Culture and Treatment

The ovarian GCs were isolated from healthy porcine follicles via syringe aspiration. Cells were rinsed with prewarmed PBS and subsequently cultured in DMEM/F12 (Gibco, Carlsbad, CA, USA) medium supplemented with 10% FBS (Ozfan, Nanjing, China) and 1% PS (Gibco, Carlsbad, CA, USA). Cultures were maintained in a humidified incubator at 37 °C with 5% CO_2_. After 36 h of incubation, cells were washed with pre-warmed PBS to remove surface debris, followed by replacement with fresh culture medium. HEK-293T cells were cultured under the same conditions as ovarian GCs, except that the culture medium used was high-glucose DMEM (Gibco, Carlsbad, CA, USA). When cell density exceeded 80%, oligonucleotides or plasmids were transfected into cells using LipofectamineTM 2000 (Invitrogen, Carlsbad, CA, USA). The oligonucleotides (miR-149: UCUGGCUCCGUGUCUUCACUCCC) were synthesized by Generay (Shanghai, China).

### 2.5. RNA Isolation and Quantification

Total RNA was extracted using FreeZol Reagent (#R711-02, Vazyme, Nanjing, China) and reverse transcribed into cDNA with SweScript All-in-One Blue RT SuperMix for qPCR (#G3329, Servicebio, Wuhan, China). The qPCR was executed using 2 × Universal Blue SYBR Green qPCR Master Mix (#G3326, Servicebio, Wuhan, China). GAPDH serves as a coding gene and cytoplasmic internal control, while U6 serves as a miRNA and nuclear internal control. The primers used here are listed in Table S1.

### 2.6. Western Blotting

The Western blotting was conducted with a previously published method [14]. Proteins were separated with the FuturePAGE^®^ gel (#ET15412Gel, ACE, Changzhou, China) and transferred to PVDF membranes (Merck MilliporeSigma, Burlington, MA, USA). Antibodies used: anti-Caspase 8 (1:1000, Biossa, Beijing, China), anti-c-Caspase 3 (1:1000, Sangon, Shanghai, China) and anti-GAPDH (1:10,000, Servicebio, Wuhan, China). Bands were visualized with a SCG-W3000 PLUS (Servicebio, Wuhan, China).

### 2.7. Nuclear-Cytoplasmic Fractionation

Nuclear-cytoplasmic fractionation was separated using the Cytoplasmic & Nuclear Extraction Kit (#C510001, Sangon, Shanghai, China), and RNA was extracted from the fractionated samples by adding TRIzol reagent.

### 2.8. Plasmids and Luciferase Assay

The Caspase 8 promoter luciferase reporter vector carrying the miR-149 binding site was synthesized by Tsingke (Nanjing, China). At 48 h after transfection, detection was performed using the Dual Luciferase Reporter Assay Kit (#DL101-01, Vazyme, Nanjing, China) and Assay System (Promega, Madison, WI, USA). The relative luciferase activity was the firefly activity/renilla activity.

### 2.9. Statistics

Values are means ± SEM and are statistically tested with GraphPad v8.0 software (San Diego, CA, USA) employing the *t*-test and Mann–Whitney U test. *p* values of <0.05 was considered as statistically significant.

## 3. Results

### 3.1. Differential miRNA Screening

**Figure 1:**
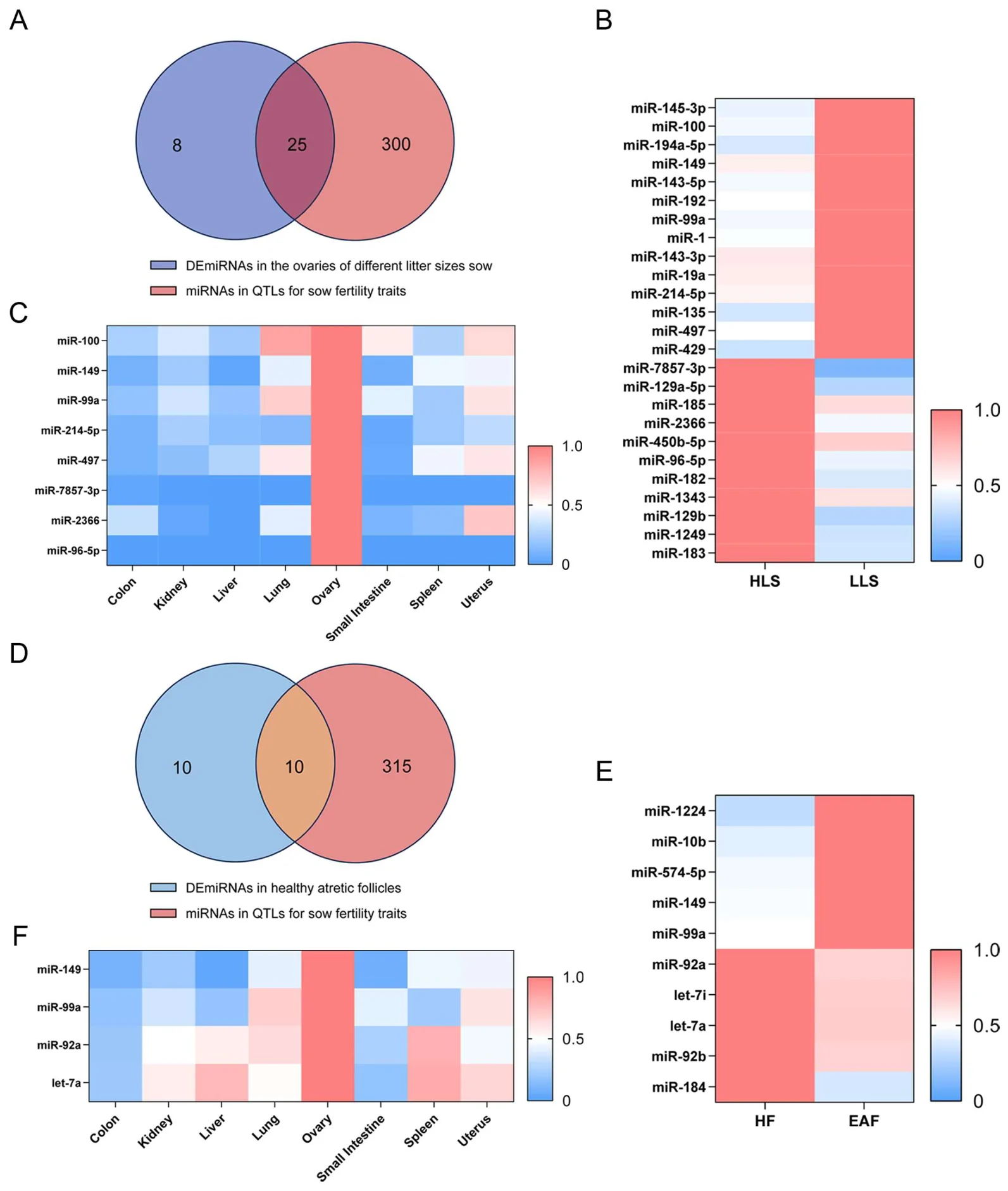
Differential miRNA screening. (**A**) Screening miRNAs located within QTLs associated with sow reproductive traits that are differentially expressed in the ovaries of different litter sizes sow. (**B**) Heatmap depicting differentially expressed miRNAs in HLS and LLS. Data were obtained from previous study [15]. (**C**) Heatmap showing the expression of miRNAs highly expressed in the ovaries across various tissues. Data were obtained from previous study [16]. (**D**) Screening miRNAs located within QTLs associated with sow reproductive traits that are differentially expressed in healthy and atresia follicles. (**E**) Heatmap depicting the expression miRNAs in HF and EAF. Data were obtained from previous study [17]. (**F**) Heatmap showing the expression of miRNAs highly expressed in the ovaries across various tissues. Data were obtained from previous study [16].

Experimentally validated QTL regions harbor fertility-associated genetic variants, so screening miRNAs within them improves the chance of identifying functionally relevant candidates. Furthermore, combining QTL positional information with miRNA-seq data provides a stringent filter to reduce false positives and enrich candidates with heritable regulatory potential, as many differentially expressed miRNAs are secondary responders rather than primary drivers. Building on this rationale, this study analyzed the miRNAs located within sow reproductive QTLs that were differentially expressed in the ovaries of sow with high litter size (HLS) and low litter size (LLS), as well as in healthy and atretic follicles, to accurately screen the key miRNAs that regulate sow reproduction. The results revealed that a total of 25 miRNAs located within sow reproductive QTLs were differentially expressed in ovarian tissues from sow with high and low litter sizes, among which 8 miRNAs exhibited high expression in ovarian tissues (Figure 1A–C). In addition, 10 miRNAs mapped to sow reproductive QTLs showed differential expression between HF and EAF, and 4 of these miRNAs were highly expressed in ovarian tissues (Figure 1D–F). In summary, this study identified several candidate miRNAs that are co-located with reproductive QTLs and exhibit differential expression during ovarian and follicular development; these miRNAs are important candidate molecules involved in the regulation of ovarian development and litter size traits in sow.

### 3.2. Identification and Functional Prediction of Reproductive Core miRNAs

**Figure 2:**
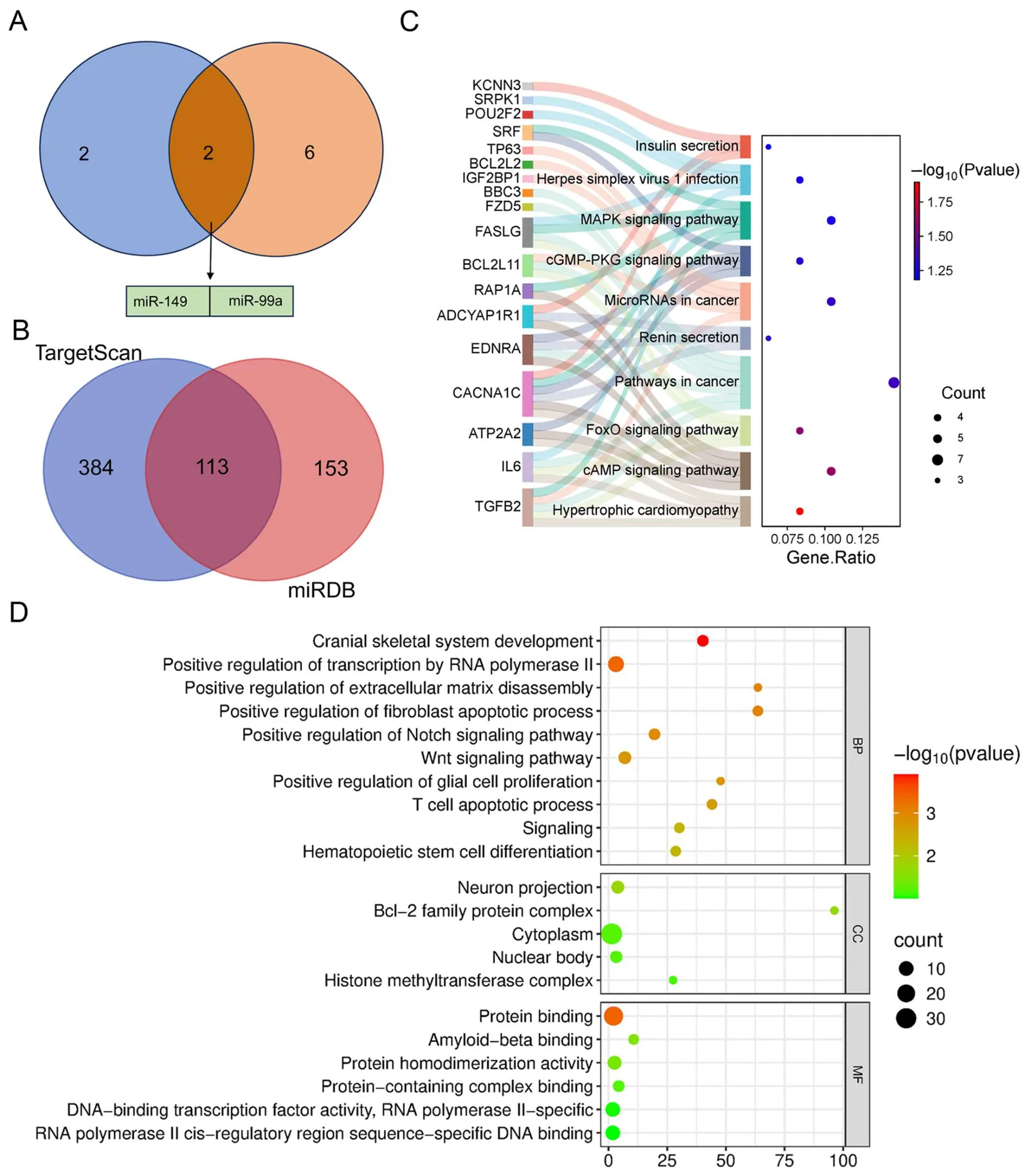
Identification and functional prediction of reproductive core miRNAs. (**A**) Screening of core miRNAs regulating sow reproductive performance. (**B**) A Venn diagram was used to illustrate the overlapping target genes of miR-149 predicted by TargetScan and miRDB. (**C**,**D**) KEGG (**C**) and GO (**D**) enrichment analysis of target genes of miR-149.

To further identify the core miRNAs that regulate sow reproduction, we conducted a secondary screening of the candidate miRNAs obtained above and identified a total of two miRNAs (miR-149 and miR-99a) that simultaneously met the following criteria: they were located within QTLs for sow reproductive traits, were differentially expressed in the ovaries of sow with HLS and LLS and in healthy atresia follicles, and were highly expressed in ovarian tissue (Figure 2A). Among these, miR-149 is located within the QTLs for litter size (Table S2), so we selected miR-149 as the subject for subsequent studies.

miR-149 target genes were predicted using TargetScan and miRDB, respectively. The two prediction tools identified 497 and 266 candidate target genes, respectively, with 113 common target genes in their intersection (Figure 2B). Functional enrichment analysis of these genes revealed that, according to KEGG pathway enrichment results, these genes were enriched in multiple signaling pathways closely related to animal reproduction, including the cAMP, FoxO, cGMP-PKG, and MAPK signaling pathway (Figure 2C, Table S3). GO functional annotation results showed that genes are involved in processes such as gene transcription regulation, protein activation, cell proliferation, and apoptosis (Figure 2D, Table S4). In summary, the miR-149 might modulate granulosa cell function via these pathways, and additional functional experiments are required to define its exact role in sow reproductive performance.

### 3.3. Characterization of miR-149

**Figure 3:**
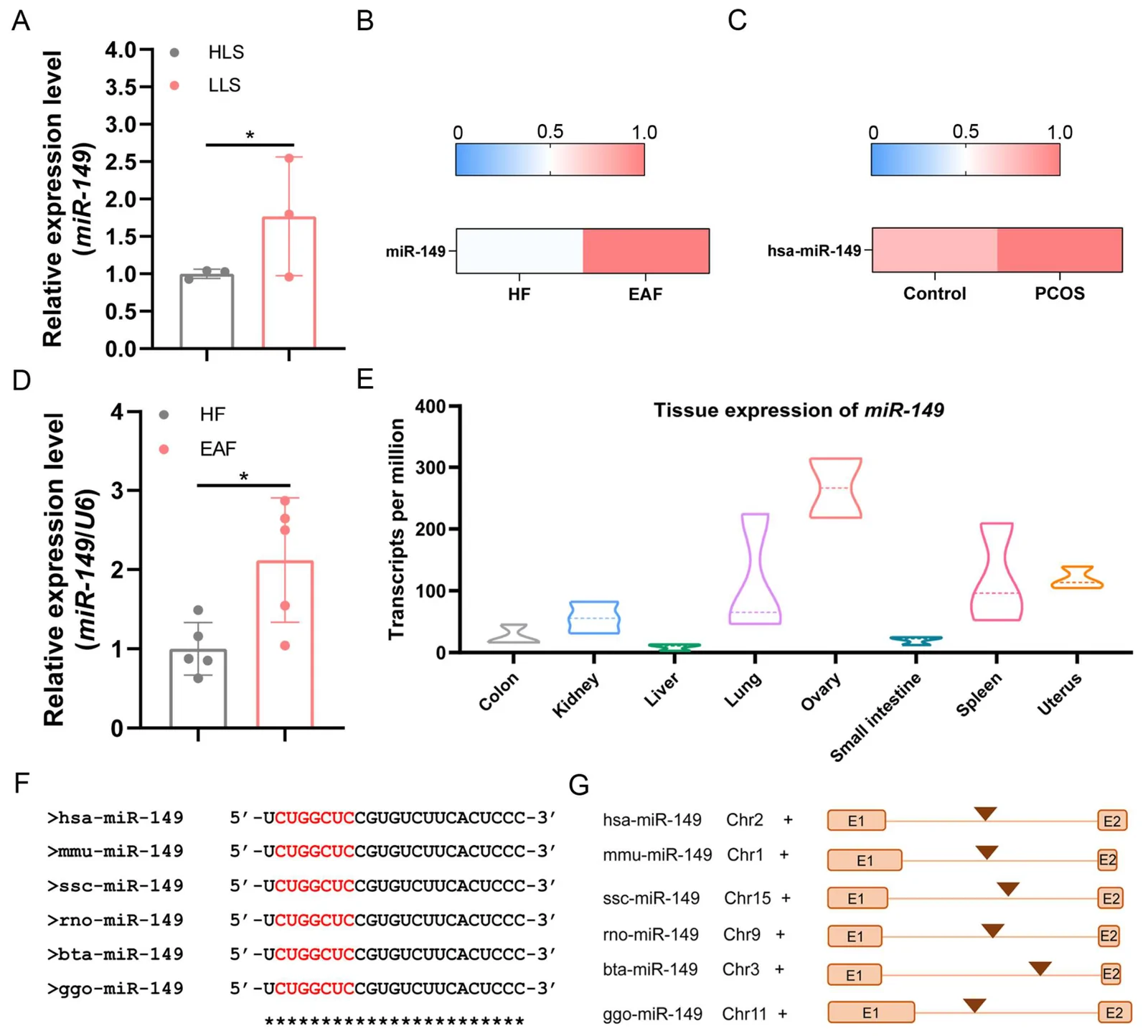
Characterization of miR-149. (**A**) Expression of miR-149 in HLS and LLS ovaries. Data were obtained from previous study [15]. (**B**) Heatmap of miR-149 expression in HF and EAF [17]. (**C**) Heat maps showing miR-149 expression in normal and PCOS ovary tissue [18]. (**D**) The levels of miR-149 in ovarian GCs of HF and EAF. *n* = 5. (**E**) Tissue expression patterns of the miR-149 in pig. The dataset was retrieved from prior research [16]. (**F**) Conservation of miR-149 across different species. Red fragments correspond to the seed region of miR-149. (**G**) Genomic structure of miR-149. The host gene GPC1 harboring miR-149 in pigs and other species is shown in the figure, which displays the genomic location of the miR-149 host gene. “E” represents exon. Values are means  ±  SEM. *, *p* < 0.05.

Previous transcriptomic sequencing results showed that miR-149 expression was upregulated in the ovaries of sow with low litter sizes, in atretic follicles of pigs, and in human polycystic ovary syndrome (PCOS) [15,17,18] (Figure 3A–C). Further qPCR validation confirmed that the expression level of miR-149 was markedly higher in atretic follicles than in healthy follicles (Figure 3D). In addition, miR-149 exhibited predominant ovarian expression, with extremely low abundance in the liver and small intestine (Figure 3E). Conservation analysis showed that the mature sequence of miR-149 (UCUGGCUCCGUGUCUUCACUCCC) and its seed region (CUGGCUC) were highly identical among various vertebrate species (Figure 3F). Moreover, miR-149 was characterized as an intronic miRNA in vertebrates, which is located within the intron of its host gene GPC1 (Figure 3G).

### 3.4. miR-149 Promotes the Expression of Apoptosis-Related Gene Caspase 3 in Ovarian GCs

**Figure 4:**
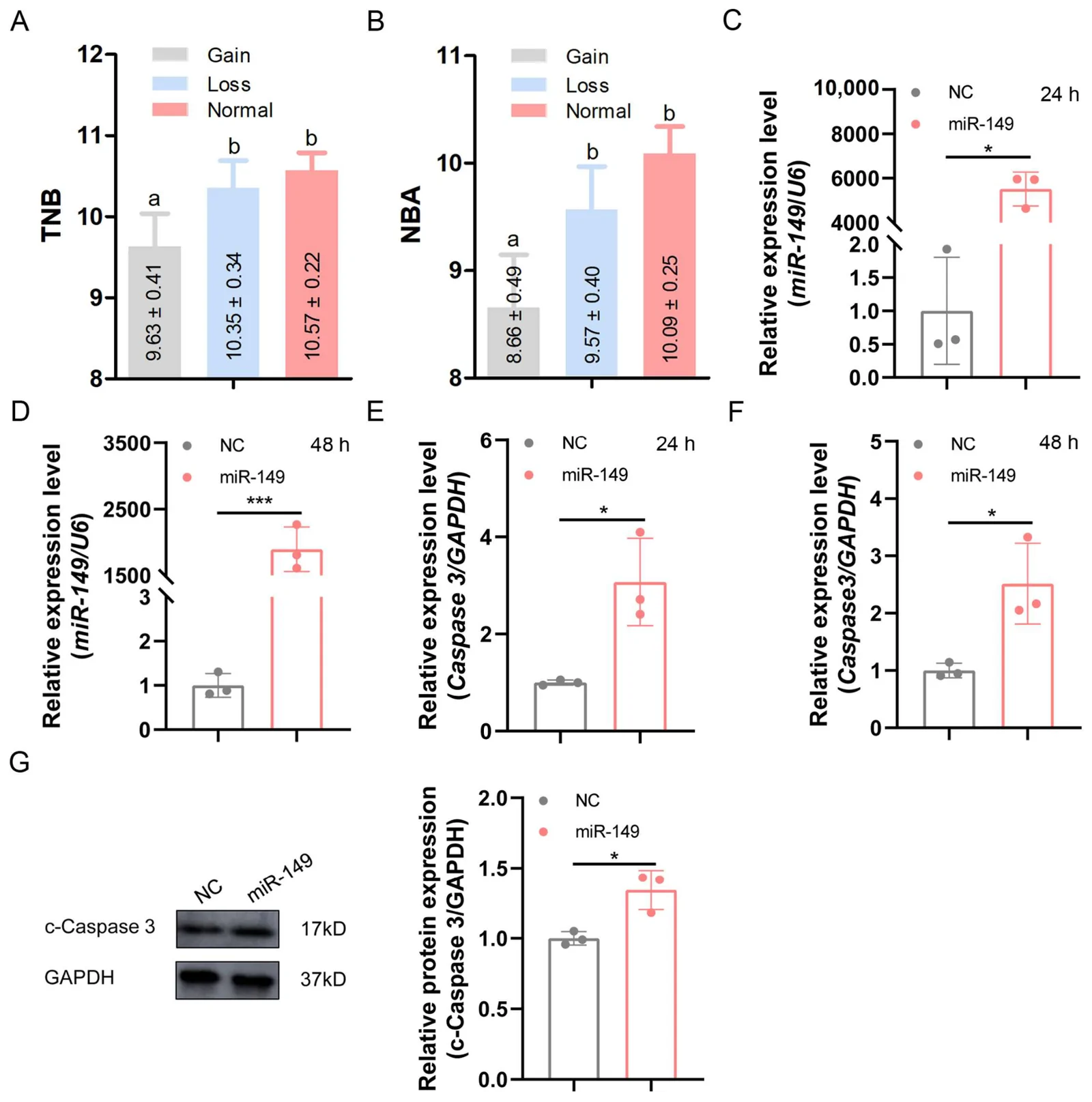
miR-149 promotes the expression of apoptosis-related gene Caspase 3 in ovarian GCs. (**A**,**B**) Effects of copy number variations in the miR-149 promoter region on TNB (**A**) and NBA (**B**) traits. Different lowercase letters (a, b) above bars indicate significant differences between groups (*p* < 0.05). Groups sharing the same letter are not statistically different. Data were obtained from previous study [19]. (**C**,**D**) Detection of miR-149 overexpression efficiency. 24 h (**C**), 48 h (**D**). NC represents miRNA negative control mimic. (**C**) Use the Mann–Whitney U test. Data were normalized to U6 miRNA levels. *n*  =  3. (**E**,**F**) The mRNA expression levels of Caspase 3 in ovarian GCs overexpressing miR-149. *n*  =  3. 24 h (**E**), 48 h (**F**). (**G**) The c-Caspase 3 protein levels in ovarian GCs after treated with miR-149. *n* = 3. *, *p*  <  0.05; ***, *p*  <  0.001.

miR-149 is located within the QTLs associated with sow reproductive traits this genomic region covers multiple litter size-related traits (Table S2). Copy number variations in the genomic region adjacent to miR-149 significantly reduce the total number born (TNB) and number born alive (NBA) in sow [19], implying that miR-149 negatively modulates sow reproductive performance (Figure 4A,B).

The GCs apoptosis is the primary cause of follicular atresia, and c-Caspase 3 serves as a key marker of cell apoptosis. To explore the role of miR-149 in sow follicular atresia, miR-149 was overexpressed in ovarian GCs. The results showed that miR-149 expression was extremely significantly elevated (Figure 4C,D). Both the mRNA and protein level of Caspase 3 were markedly increased in miR-149-overexpressed ovarian GCs (Figure 4E–G). Collectively, gain-of-function experiments demonstrated that miR-149 participates in follicular atresia and impairs sow reproductive performance by promoting ovarian GCs apoptosis.

### 3.5. miR-149 Promotes the Expression of Caspase 8 in Ovarian GCs

**Figure 5:**
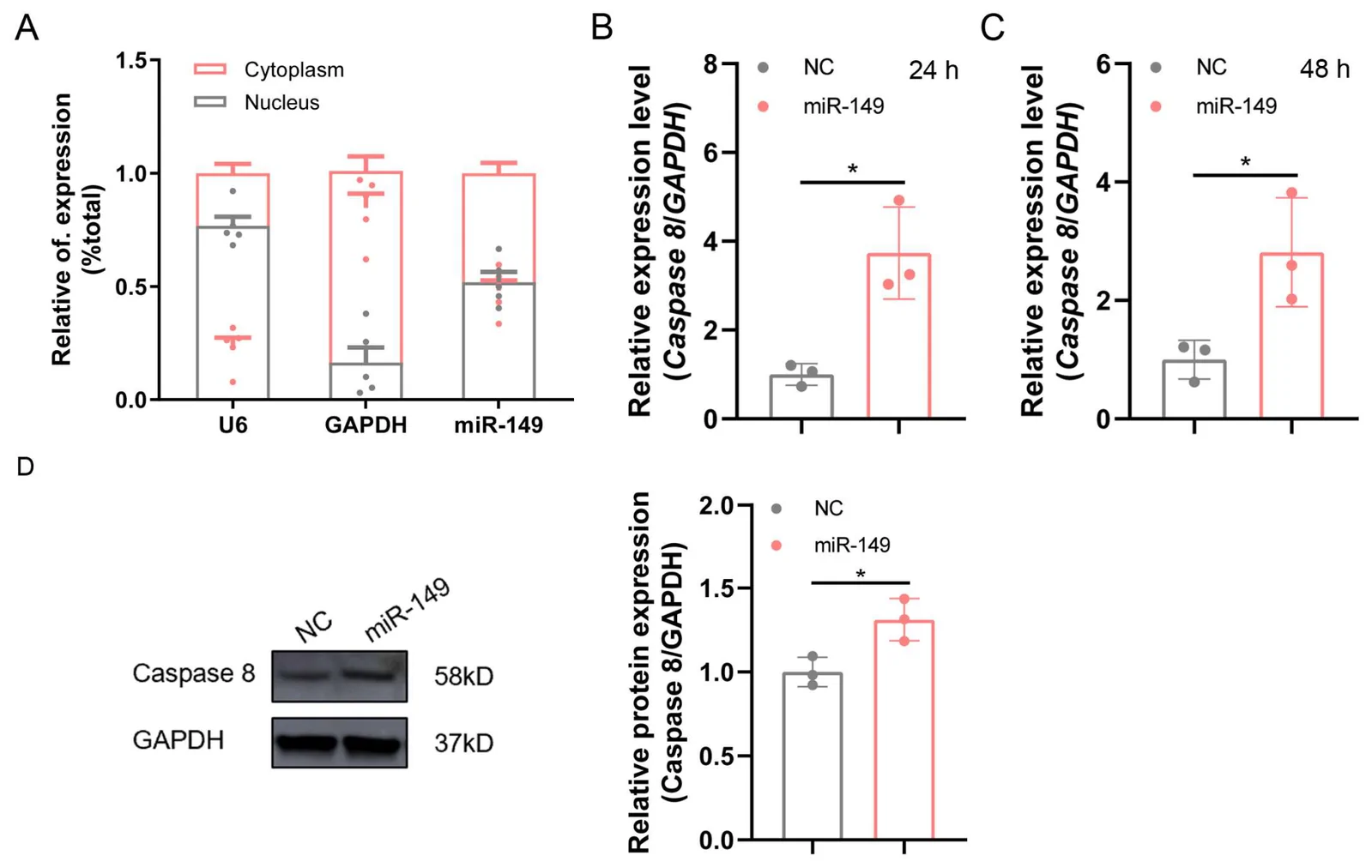
miR-149 promotes the expression of Caspase 8 in ovarian GCs. (**A**) Subcellular localization analysis of miR-149 in ovarian GCs. The expression levels of miR-149 in nuclear and cytoplasmic of ovarian GCs were determined via qPCR. U6 and GAPDH were used as the nuclear and cytoplasmic reference genes, respectively. *n* = 5. (**B**,**C**) The mRNA expression of Caspase 8 in miR-149 mimic-treated ovarian GCs was quantified via qPCR. 24 h (**B**), 48 h (**C**). Data were normalized to GAPDH mRNA levels. *n*  =  3. (**D**) The Caspase 8 protein levels in ovarian GCs after treated with miR-149. *n* = 3. *, *p*  <  0.05.

The subcellular localization of miRNAs determines their functional mechanisms [20]. To clarify the subcellular distribution characteristics of miR-149, nuclear and cytoplasmic RNAs were isolated from ovarian GCs. The results showed that miR-149 was expressed in both the nucleus and cytoplasm (Figure 5A), implying that it may regulate target gene expression via the RNA activation (RNAa) mechanism. The Caspase 8 acts as a crucial initiator in the death receptor-mediated apoptotic pathway and functions upstream of Caspase 3 [21]. We detected the effect of miR-149 overexpression on Caspase 8 expression. The results demonstrated that miR-149 significantly upregulated expression level of Caspase 8 in ovarian GCs (Figure 5B–D). Collectively, these results indicate miR-149 functions positive regulator of Caspase 8.

### 3.6. miR-149 as saRNA to Activate Caspase 8 Transcription in Ovarian GCs

**Figure 6:**
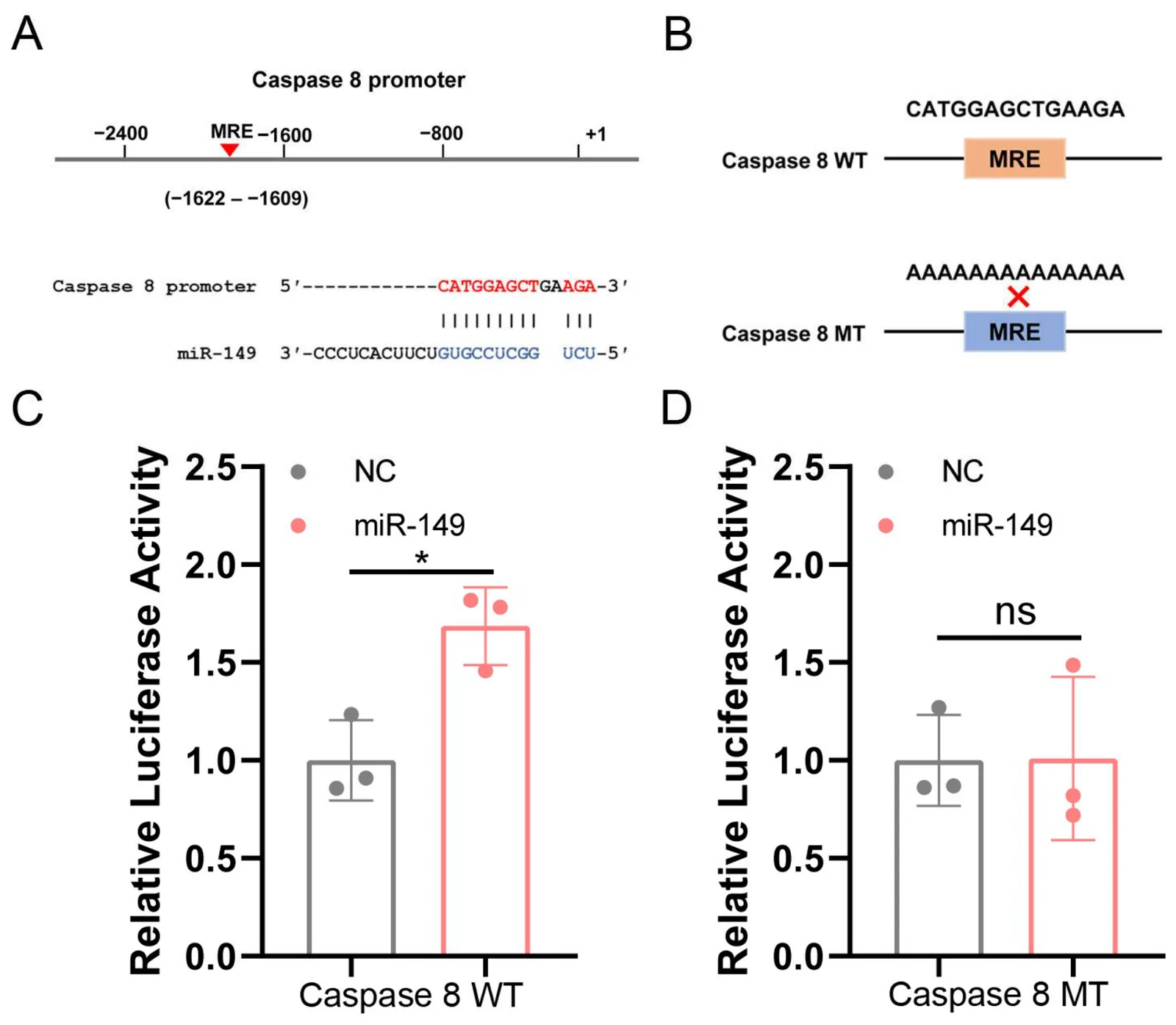
miR-149 activates Caspase 8 transcription. (**A**) Schematic diagram of the interaction between miR-149 and the Caspase 8 promoter. (**B**) Schematic illustration of wild-type and mutant Caspase 8 promoters carrying miR-149 binding sites. (**C**) After transfection of Caspase 8 WT reporter vector and miR-149 into HEK-293T cells, luciferase activity was detected. *n* = 3. (**D**) After transfection of Caspase 8 MT reporter vector and miR-149 into HEK-293T cells, luciferase activity was detected. *n* = 3. Values are means  ±  SEM. *, *p*  <  0.05; ns, no significant.

Nuclear miRNAs exert RNAa functions by binding to the promoter regions of target genes [22]. Therefore, RNAhybrid was used to predict the potential miRNA response element (MRE) within the Caspase 8 promoter. The results revealed that one putative MRE for miR-149 was located at −1622 to −1609 bp of the Caspase 8 promoter (Figure 6A). To verify whether miR-149 activates Caspase 8 expression via this MRE, we generated luciferase reporter plasmids carrying wild-type or mutated miR-149 binding sites of the Caspase 8 promoter (Figure 6B). Dual-luciferase assay results showed that miR-149 overexpression markedly enhanced the luciferase activity of the wild-type Caspase 8 promoter, but exerted no significant effect on the activity of the MRE-mutant promoter (Figure 6C,D). These data indicated that miR-149 enhances Caspase 8 promoter activity by directly targeting this MRE motif. In conclusion, miR-149 functions as saRNA to activate the transcription of Caspase 8.

## 4. Discussion

Over 99% of ovarian follicles in mammals experience atresia and degradation across distinct developmental phases, and only a small number of follicles can eventually mature and ovulate [23]. Previous studies have confirmed that dysregulated expression of miRNAs is closely linked to porcine follicular atresia, thereby modulating sow reproductive performance. Nevertheless, most existing studies screen candidate molecules merely based on single transcriptomic data, leading to a wide screening range and difficulty in accurately identifying the key genes that directly regulate reproductive traits. In this study, by integrating multiple transcriptomic datasets with reproductive trait QTL interval mapping, miR-149 was identified as a core regulator of sow reproductive performance. We further systematically demonstrated that miR-149 modulates ovarian GCs apoptosis and mediates follicular atresia via the nuclear RNAa mechanism, thereby exerting a negative regulatory effect on sow litter performance. The above results lay a theoretical foundation for developing molecular regulation technologies to inhibit follicular atresia and improve sow reproductive efficiency.

miR-149 is dysregulated in a variety of human diseases, including cancer, skin diseases, and cardiovascular diseases [24,25,26]. In pigs, miR-149 is differentially expressed during physiological processes such as follicular development, sexual maturation, and fat deposition, suggesting that miR-149 has broad biological functions [17,27,28]. miRNAs typically exert their functions through their downstream target genes. We used TargetScan and miRDB to predict the target genes of miR-149 and identified 113 common target genes. These genes are enriched in signaling pathways such as the cAMP, FoxO, cGMP-PKG, and MAPK signaling pathway. It is worth noting that these signaling pathways have been shown to regulate reproduction in animals [29,30,31,32]. In addition, these genes are involved in core biological processes that affect reproduction, such as gene transcription regulation, protein activation, cell proliferation, and apoptosis. It can therefore be inferred that miR-149 may be involved in regulating reproductive traits in sow by synergistically modulating multiple reproduction-related signaling pathways.

Tissue expression profiling serves as an important basis for predicting the biological functions of genes and elucidating their tissue-specific regulatory mechanisms [33]. miR-149 was significantly highly expressed in sow ovarian tissues, suggesting that it may play an essential role in maintaining ovarian physiological functions and regulating follicular development. To further clarify the genomic characteristics and evolutionary conservation of miR-149, we systematically analyzed its sequence features, host gene attribution, and genomic localization. The results confirmed that miR-149 is an intronic miRNA located within the intron 1 of the porcine GPC1 gene. Its core sequence, host gene relationship, and intronic localization pattern are highly conserved across different species. Accumulating evidence has demonstrated that intronic miRNAs often form synergistic or interactive regulatory networks with their host genes to jointly execute specific biological functions [34,35]. Previous studies have demonstrated that GPC1 is involved in regulating cell proliferation and angiogenesis [36,37,38,39], both of which are key regulatory processes in follicular development [40,41]. Accordingly, the genomic localization characteristics of miR-149 identified in this study provide a theoretical foundation and research direction for further exploring the synergistic regulatory mechanism between miR-149 and its host GPC1, as well as refining the miR-149-mediated regulatory pathway of follicular development. In addition, qPCR validation revealed that miR-149 was markedly upregulated in atretic follicles, confirming a positive correlation between its expression level and the progression of follicular atresia. Notably, previous studies have verified that copy number variation in the miR-149 genomic region significantly affects core reproductive traits such as TNB and NBA in sow, and miR-149 is mapped within key reproductive trait QTL intervals [19]. These findings further support the negative regulatory role of miR-149 in sow reproductive performance from a genomic genetic perspective. Human-based clinical studies have shown that miR-149 mutations are significantly associated with recurrent spontaneous abortion [42,43,44,45,46]. Elevated miR-149 expression is also detected in patients with recurrent spontaneous abortion [47,48]. Functional experiments confirm that miR-149 overexpression induces cell apoptosis and suppresses cell viability [48], suggesting that this molecule may also play a potential role in maintaining human reproductive homeostasis.

Canonical miRNAs typically exert inhibitory functions by complementary binding to the 3′UTR of target genes, which suppresses mRNA stability or protein translation and thereby participates in various biological processes [49]. For instance, miR-205a [50], miR-454-3p [51], and miR-221 [52] all function through this classical regulatory mode. Unlike these classical mechanisms, our results revealed that miR-149 is distributed in both the nucleus and cytoplasm of ovarian GCs, implying that it may play a positive transcriptional regulatory role through the nuclear-located RNAa mechanism. Further experimental validation confirmed that miR-149 specifically binds to the promoter of Caspase 8 and activates its transcription. This mechanism is consistent with the characteristic of miRNAs acting as saRNAs to activate transcription by binding to the promoter regions of target genes [53,54]. In addition, studies on miRNA acting as saRNA have shown that when miRNAs mediate regulation via saRNAs, there is a time lag in their effects; the regulatory effects are primarily observed at 48 h, with no significant regulatory effects at 24 h [8,20]. The results of this study show that transfecting ovarian GCs with miR-149 significantly upregulates Caspase 8 mRNA expression at both 24 h and 48 h post-transfection. Given that miR-149 is expressed in both the nucleus and the cytoplasm, we speculate that the regulatory mechanism of miR-149 on Caspase 8 is not limited to the transcriptional level. Previous studies have verified that certain miRNAs can specifically bind to the 5′UTR regions of target transcripts. Via multiple mechanisms including mRNA stabilization and relief of translational repression, these miRNAs increase the mRNA abundance and protein levels of target genes, thereby exerting positive regulatory activation on target gene expression [14,55,56,57]. Our predictions revealed that the 5′UTR region of the Caspase 8 gene contains multiple potential binding sites for miR-149 (Table S5), suggesting that miR-149 may also stabilize Caspase 8 mRNA through post-transcriptional mechanisms, thereby upregulating its expression. In addition, we analyzed the potential binding of miR-149 to the Caspase 8 promoter and 5′UTR in humans and mouse (Tables S6 and S7). miR-149 binding sites were predicted in both of these regions, suggesting that this regulatory mechanism of miR-149 may be conserved across species.

Caspase 8 and Caspase 3 are key members of the caspase family. The Caspase 8 acts as a critical initiator in the death receptor-mediated apoptotic pathway [58], while c-Caspase 3 serves as the essential executioner of cell apoptosis [59]. Dysregulated transcription of Caspase 8 can disrupt the activity of the death receptor-mediated apoptotic cascade [60]. In this study, miR-149 functions as a saRNA to activate Caspase 8 transcription and further trigger the death receptor apoptotic pathway. Accumulating evidence has demonstrated that GCs apoptosis is the direct cause of follicular atresia, and multiple miRNAs mediate this process by regulating GCs apoptosis, such as miR-484 [61], miR-6881-3p [62], miR-29c [63], miR-361-5p [10], and miR-1275 [12]. Consistent with these reports, during follicular atresia, miR-149 expression levels increase, and it acts as an apoptotic factor. Nevertheless, there are notable mechanistic distinctions between them. The previously reported miRNAs exert their functions by binding to the 3′UTR of target genes to reduce target mRNA abundance. By contrast, miR-149 binds to the promoter region of Caspase 8 and enhances its transcription, thereby triggering GCs apoptosis.

Overall, the present findings reveal a novel mechanism underlying the transcriptional regulation of Caspase 8 and define a new regulatory pathway controlling ovarian GCs apoptosis. This study further complements the functional evidence of miR-149 in regulating sow reproductive traits at the cellular level. In future studies, in vivo overexpression and knockdown models can be used to verify the biological function of miR-149, and its upstream and downstream regulatory molecules can be further explored to refine the regulatory network governing sow follicular development and reproductive performance. These efforts will provide comprehensive theoretical references and valuable molecular markers for molecular breeding of high-yield sow and the prevention and treatment of reproductive disorders.

## 5. Conclusions

This study systematically revealed the function and molecular mechanism by which miR-149 acts as a negative regulator affecting sow reproductive performance. We clarified that miR-149 mediates follicular atresia by activating the Caspase 8/Caspase 3 apoptotic pathway via the RNAa mechanism. These findings provide novel targets and theoretical support for investigating the molecular regulatory mechanisms underlying sow reproductive traits.

## Data Availability

All data generated or analyzed during this study are included in the published content of this article and its Supplementary Materials files.

## Supplementary Materials

The following supporting information can be downloaded at: https://www.mdpi.com/article/10.3390/ani16172663/s1, Table S1: Primers for qPCR; Table S2: The QTLs associated with reproductive traits containing miR-149; Table S3: The KEGG analysis showing enriched signaling pathways of miR-149 target genes; Table S4: The GO enrichment results of miR-149 target genes; Table S5: The potential miR-149 binding sites in the 5′UTR of the caspase 8; Table S6: The potential binding sites for human miR-149 and Caspase 8; Table S7: The potential binding sites for mouse miR-149 and Caspase 8; Figure S1: The uncropped images of Figure 4G; Figure S2: The uncropped images of Figure 5D.

## Abbreviations

The following abbreviations are used in this manuscript:

QTL	Quantitative trait loci
GCs	Granulosa cells
saRNA	Small activating RNA
c-Caspase3	Cleaved Caspase3
miRNAs	microRNAs
UTR	Untranslated region
P4	Progesterone
E2	17β-estradiol
HF	Healthy follicles
EAF	Early atresia follicles
HLS	High litter size
LLS	Low litter size
TNB	Total number born
NBA	Number born alive
PCOS	Polycystic ovary syndrome
RNAa	RNA activation
MRE	miRNA response element
