# Supplementary material for: miR-149 Promotes Apoptosis of Ovarian Granulosa Cells Through Inducing Caspase 8 Expression
Source: Animals (Basel). 2026 Aug 25;16(17):2663. doi: 10.3390/ani16172663 (PMC13564309; doi:10.3390/ani16172663)

Figure S1: The uncropped images of Figure 4G

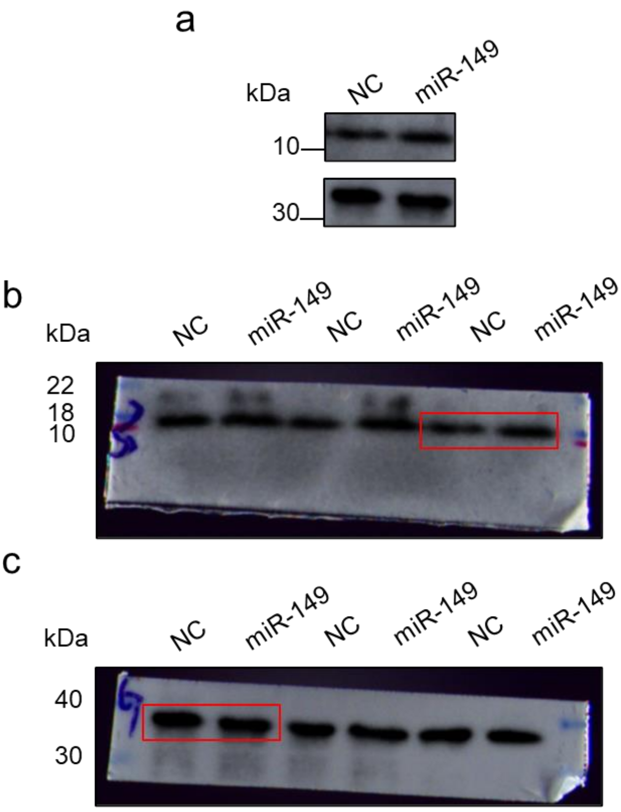

Figure S2: The uncropped images of Figure 5D

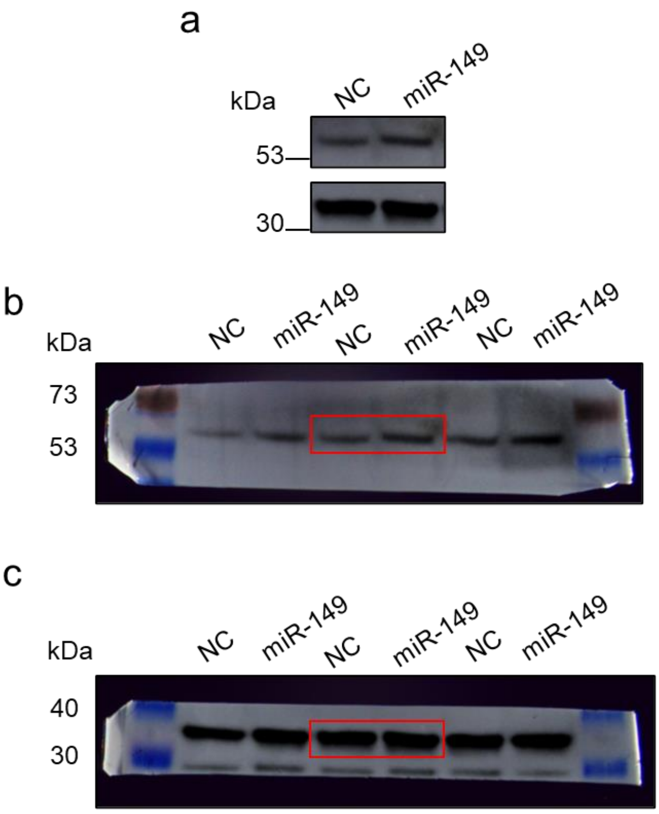

Supplement: Supplementary file 1 [file animals-16-02663-s001.zip › Supplementary Figures.pdf]
